# Continuation of Teletherapy After the COVID-19 Pandemic: Survey Study of Licensed Mental Health Professionals

**DOI:** 10.2196/32419

**Published:** 2022-06-01

**Authors:** Rashmi Gangamma, Bhavneet Walia, Melissa Luke, Claudine Lucena

**Affiliations:** 1 Department of Marriage and Family Therapy Falk College of Sport and Human Dynamics Syracuse University Syracuse, NY United States; 2 Department of Public Health Falk College of Sport and Human Dynamics Syracuse University Syracuse, NY United States; 3 Department of Counseling & Human Services School of Education Syracuse University Syracuse, NY United States

**Keywords:** teletherapy, relational teletherapy, teletherapy predictors, postpandemic teletherapy, mental health, telemedicine, COVID-19, telehealth

## Abstract

**Background:**

The use of teletherapy has exponentially increased in the context of the ongoing COVID-19 pandemic. Studies on teletherapy documented substantial benefits of accessibility and convenience even before the start of the pandemic. Although recent studies show that this modality of therapy delivery is here to stay, few have studied who will most benefit from this trend.

**Objective:**

In this paper, we report predictors of continued teletherapy usage in a sample of licensed mental health professionals in the United States during a time period when pandemic-related restrictions began diminishing. As such, it is one of the first studies to examine factors related to continued benefits of teletherapy postpandemic.

**Methods:**

Participation from licensed mental health professionals was sought on listservs of national organizations of multiple mental health organizations. Data were collected via an anonymous link to a survey on Qualtrics between January 2021 and April 2021. Participants responded to questions on therapist demographics, practice setting, experiences of shifting to teletherapy, perspectives on continued use of teletherapy, and their client characteristics. Findings related to client characteristics that predicted continued teletherapy usage are presented here.

**Results:**

A total of 186 individuals consented to participate in the survey, with a final sample of 114 with complete data. A majority of participants identified as female (92/114, 80.7%), White (94/114, 82.5%), and having a master's degree (75/114, 65.5%) from a nationally accredited program (106/114, 93%). Data were analyzed using heteroskedastic regression modeling with client-related factors as predictors. Two models were run with and without distance travelled by clients as a control variable. Model estimates from both models showed that continued use of teletherapy postpandemic was predicted by the following factors: higher percentage of clients from rural areas, younger and older adult clients, clients with Medicare, and clients with marginalized gender and religious/spiritual identities. Significantly, having a higher percentage of clients from lower socioeconomic status, a higher percentage of those with Medicaid coverage, and a higher percentage of couples and families as clients predicted decreased use of teletherapy postpandemic.

**Conclusions:**

Findings from the study suggest that while some groups of clients are more likely to continue to receive benefits of teletherapy, vulnerable groups such as those in lower socioeconomic conditions, Medicaid beneficiaries, and those who seek couple and family therapy may be less likely to be served by it. These differences point to a need to address factors driving telehealth care disparities such as access to technology, housing, and childcare issues, as well as the need for continued training for licensed professionals.

## Introduction

The COVID-19 pandemic and subsequent social measures drastically impacted society [1], shifting education, work, health care [2,3], and mental health [4]. Telemental health, referred to as teletherapy, has been used over the past 20 years [5] with demonstrated effectiveness [6,7]. Teletherapy refers to the use of electronically based communication such as videoconferencing, telephone calls, and mobile apps to provide access to mental health services, typically across distances [8]. Rapid legislative changes, training, and guidelines resulted in an exponential increase in teletherapy when compared to prepandemic levels [9,10]. The increase in relational teletherapy (teletherapy with couples and families) has been particularly important given increased risks for distress, anxiety, grief/loss, substance abuse, and family violence in children [11] and adults [12-14] during the pandemic. Before the COVID-19 pandemic, scholars contended that historically underserved populations derived more benefits from the flexibility and accessibility of teletherapy [15,16]. As COVID-19–related restrictions are lifted, teletherapy will remain part of the mental health landscape [17]. However, given the existing challenges of the need for training, technological advances, and other barriers to effective use [8,18,19], we are yet to understand whether teletherapy will be accessible equitably postpandemic.

In this paper, we present findings from a study on predictors of continued teletherapy practice postpandemic from a sample of licensed mental health practitioners. Specifically, our research question was “What factors of therapist practice predict their intention for continued use of teletherapy practice postpandemic?” Existing literature suggests that distance from services, client profile [15,16], and vulnerability of selected client populations [6-8,18-20] may influence provision of teletherapy. Clarifying predictors would strengthen recent research on therapists’ experiences transitioning to the use of telehealth [18] and may assist in identifying factors in disparities in telehealth care postpandemic.

## Methods

### Recruitment

Participation was open to licensed mental health professionals who were currently providing teletherapy. Upon institutional review board approval, a link to an anonymous Qualtrics survey was posted on multiple listservs including the American Association for Marriage and Family Therapy, the American Counseling Association, as well as professional groups for social workers. Data were gathered between January 2021 and April 2021, when increased vaccinations were driving gradual removal of public health reductions [20]. Survey questions included therapist demographics, practice setting, experiences of shifting to teletherapy, perspectives on continued teletherapy use, and client characteristics. No incentives were provided; instead, a donation was made to a nonprofit chosen by participants. A total of 186 individuals consented to participate in the survey, with a final sample of 114 with complete data.

### Ethics Approval

This study received ethics approval from Syracuse University’s Institutional Review Board (IRB #20-310).

### Statistical Analysis

Descriptive statistics and regression analyses were conducted using Stata software (version 14; StataCorp LLC) [21]. A residual plot revealed increasing standard deviation of residuals in the independent variables (ie, heteroskedasticity). Given that errors were normally distributed and mean and variance functions were correctly specified, we ran hetregress regression models with maximum likelihood estimator [21]. Using G*Power power analysis, setting a medium effect size with 10 predictors in our model, we determined that our final sample of 114 was sufficient for regression analysis [22].

## Results

Participants were from 27 states in the United States, with a majority identifying as female (92/114, 80.7%), White (94/114, 82.5%), and with a master's degree (75/114, 65.5%) from a nationally accredited program (106/114, 93%). Less than half of participants (45/114, 39.5%) reported prepandemic experience practicing teletherapy. Table 1 shows other practice profiles of participants and Table 2 shows client profile factors used as independent variables in the regression models.

Table 3 shows coefficient values of regression models run without and with control for distance travelled by clients (models 1 and 2, respectively). We controlled for distance from a health setting in model 2 to limit multicollinearity and increase robustness of estimates. Both models were estimated with therapist gender as a cluster variable.

Among factors examined, statistically significant predictors were (1) higher percentage of clients living further from a metro area, particularly those in rural areas (β=38.578, *P*<.01), (2) higher percentage of clients who are younger (<30 years; β=.186, *P*<.001) or older (65-80 years; β=.634, *P*<.001), (3) higher percentage of clients who identified with a minoritized gender (β=.223, *P*<.001) and religious/spiritual identity (β=.153, *P*<.001), and those with disabilities (β=.399, *P*<.001), and (4) higher percentage of clients with Medicare (β=.457, *P*<.001).

Conversely, therapists for whom couples/families were >75% of their caseload were less likely to continue teletherapy compared to therapists with caseloads of couples/families <25% (β=19.876, *P*<.001), 25%-50% (β=32.040, *P*<.001) and 50%-75% (β=28.927, *P*<.001). Similarly, therapists with a higher percentage of clients from lower socioeconomic backgrounds (β=–.285, *P*<.001) and a higher percentage of clients with Medicaid coverage (β=–.143, *P*<.05) were less likely to continue teletherapy postpandemic.

**Table 1:** Practice profiles of participants (N=114).

Practice profile of participants		Participants, n (%)
**Type of license**		
	Marriage and family therapy	77 (67.5)
	Mental health counselor	21 (18.2)
	Clinical social work	5 (4.4)
	Clinical psychologist	4 (3.5)
	Other	7 (6.1)
**Geographical location**		
	Large metro	36 (31.9)
	Medium metro	32 (28.3)
	Small metro	27 (23.9)
	Rural area	6 (5.3)
	Small town	5 (4.4)
**Distance travelled by clients**		
	<25 miles	98 (85.8)
	25-50 miles	13 (11.5)
	>50 miles	3 (2.4)

**Table 2:** Descriptive of client profile factors used in regression models.

Client profile			Average percentage^a^
**Age group (years)**			
	<30	44.05	
	30-49	38.75	
	50-64	10.83	
	65-80	4.20	
	>80	0.34	
**Gender**			
	Female	56.42	
	Male	34.81	
	Nonbinary/gender expansive	5.19	
	Transgender	4.81	
	Other	1.39	
**Marginalized identities**			
	Marginalized gender identities	15.22	
	Marginalized sexual identities	17.79	
	Marginalized racial/ethnic identities	26.22	
	Marginalized religious/spiritual identities	10.01	
	Lower socioeconomic status groups	28.38	
	Having a disability	15.91	
	Veterans	5.96	
**Payer mix**			
	Medicaid	13.01	
	Medicare	4.42	
	Private health insurance	27.81	
	Veterans Health Care	2.19	
	Self-pay	43.71	
	Other	8.63	
**Percentage of couples and families in case load**			
	<25%	42.98	
	25%-50%	0.34	
	50%-75%	11.40	
	>75%	12.28	

^a^Absolute values are unavailable because the average percentage was calculated for each group.

**Table 3:** Regression model of client factors predicting therapists’ postpandemic teletherapy usage.

Factors			Model 1 (n=94)					Model 2 (n=94)		
			Coefficient		SE		Coefficient			SE
**Practice setting**										
	Fringe large metro	6.792		0.436		9.670			0.499	
	Medium metro	7.495^a^		3.418		5.545^b^			1.876	
	Small metro	6.620^a^		3.960		5.401			0.928	
	Micropolitan	16.804^a^		2.804		15.939^a^			3.028	
	Rural	39.843^c^		1.970		38.578^c^			2.079	
**Percentage of couples and families in case load**										
	<25%	25.291^a^		3.518		19.876^a^			2.993	
	25%-50%	39.158^a^		29.207		32.040^a^			9.333	
	50%-75%	35.416^a^		5.746		28.927^a^			4.351	
**Client age (years)**										
	<30	0.213^a^		16.047		0.186^a^			7.052	
	30-49	0.277^a^		28.157		0.226^a^			5.083	
	51-64	–0.215		–0.655		–0.135			–0.365	
	65-80	0.661^c^		2.468		0.634^a^			2.961	
**Percentage of clients with marginalized identities**										
	Racial/ethnic identities	0.089		0.921		0.134			1.129	
	Sexual identities	0.005		0.033		0.009			0.079	
	Gender identities	0.276^a^		4.766		0.223^a^			6.154	
	Religious/spiritual identities	0.109^c^		2.069		0.153^b^			1.855	
	Lower socioeconomic status	–0.341^a^		–3.879		–0.285^a^			–3.264	
	Disability	0.417^a^		6.261		0.399^a^			3.734	
**Client payment modality**										
	Medicaid	–0.066		–0.871		–0.143^b^			–1.649	
	Medicare	0.390^a^		4.139		0.457^a^			4.823	
	Private insurance	–0.071^a^		–4.344		–0.079^a^			–3.712	
	Other pay	0.148^a^		3.151		0.090^a^			2.787	
Constant			–83.033^a^		–6.727		–87.333^a^			–6.786
Insigma 2 Constant			6.068^a^		58.085		6.161^a^			54.540

^a^*P*<.001.

^b^*P*<.05.

^c^*P*<.01.

## Discussion

### Principal Findings

Results illuminate the potential types of clients most likely to continue to receive teletherapy postpandemic from licensed mental health professionals in our sample. In addition to supporting earlier literature on use of teletherapy with clients with disabilities and from rural areas [23,24], our findings suggest that younger and older adult clients, those on Medicare, and clients who identified with marginalized gender or religious/spiritual identities are most likely to continue to receive teletherapy. It is likely that legislative actions leading to waivers of restrictions and increased coverage of teletherapy [25,26] benefitted older adult clients and those with Medicare coverage. For clients with minoritized social identities who could also access teletherapy, changes during the pandemic may have highlighted the relative safety of seeking therapy via technology.

We also found that therapists were less likely to continue teletherapy when they had a higher percentage of clients from lower socioeconomic backgrounds and with Medicaid coverage or had a higher percentage of caseloads with couples and families. Given that the pandemic has disproportionately impacted those who are underresourced, decreased teletherapy usage with those with lower socioeconomic status suggests that unless structural issues of accessibility are addressed, vulnerable groups may be left behind. Studies report technological difficulties, lack of confidential space, and privacy concerns hinder relational teletherapy [27]. It is possible these barriers are indicative of a need for structural changes (eg, access to adequate housing, broadband internet, and childcare) to prevent deepening disparities. Although therapists with a higher percentage of Medicare clients were likely to continue its use, those with a greater percentage of Medicaid clients were less likely to do so. Given both Medicare and Medicaid coverage of teletherapy began at the same time, this difference may be a factor of available client resources or discrepancies in support between the two programs at state and local levels.

Another significant finding is therapists with the highest percentage of couples and families in their caseload were less likely to continue teletherapy. Although we did not ask for their reasons, this is consistent with earlier studies identifying challenges of training [8], difficulties in de-escalating, and simultaneous engagement with multiple family members [28]. Although teletherapy presents several advantages for access with partners in multiple locations or families with young children [7,18,27], COVID-19 factors related to remote work and school, limited space at home, and lack of social support may have resulted in intense situations [29] that were challenging to address via teletherapy. Studies have reiterated these challenges, including the possibility of therapist exhaustion [30], moral distress [31], split alliances [18], and lack of training and competencies in teletherapy [8]. Moving forward, competency-based training [19] and best practices for telemental health must attend to the unique challenges of working with couples and families [27] along with ways in which therapists can be better supported [32]. Further research is also needed to better differentiate therapists’ experiences with telehealth in general from their unique experiences of teletherapy during the COVID-19 pandemic [18].

### Limitations

Although this study recruited from different states and mental health disciplines, and the findings are robust, they are still exploratory and tentative. Participants self-selected to take part in the survey, and it is possible they had specific experiences that may not reflect views of the national population of therapists, limiting generalizability. Future research with a diverse sample and increased heterogeneity is needed. Doing so may result in less heteroscedastic data and extend our understanding of how aspects of the therapist, client, and practice contexts intersect.

### Conclusion

Public health concerns and health safety underscored the shift to teletherapy [33], rather than a structured or clinically sound plan to increase access with trained practitioners. As we emerge from pandemic-related restrictions, it is likely that teletherapy will continue [17]. However, few studies have examined mental health providers’ perspective on potential inequities of shifting to teletherapy [34] and the resultant disproportionate experiences of those living in underresourced communities [35]. Although access and convenience drive teletherapy use [36], our study suggests that after the pandemic, licensed professionals are less likely to continue teletherapy for clients in lower socioeconomic groups as well as for many couples and families. We contend that training clinicians and addressing structural barriers to teletherapy access may decrease deepening disparities in teletherapy provision.
